# An ‛Aukward’ Tale: A Genetic Approach to Discover the Whereabouts of the Last Great Auks

**DOI:** 10.3390/genes8060164

**Published:** 2017-06-15

**Authors:** Jessica E. Thomas, Gary R. Carvalho, James Haile, Michael D. Martin, Jose A. Samaniego Castruita, Jonas Niemann, Mikkel-Holger S. Sinding, Marcela Sandoval-Velasco, Nicolas J. Rawlence, Errol Fuller, Jon Fjeldså, Michael Hofreiter, John R. Stewart, M. Thomas P. Gilbert, Michael Knapp

**Affiliations:** 1 Molecular Ecology and Fisheries Genetics Laboratory, School of Biological Sciences, Bangor University, Bangor, Gwynedd LL57 2UW, UK; g.r.carvalho@bangor.ac.uk; 2 Natural History Museum of Denmark, University of Copenhagen, Øster Voldgade 5–7, 1350 Copenhagen K, Denmark; drjameshaile@gmail.com (J.H.); jose.samaniego@snm.ku.dk (J.A.S.C.); j.niemann@snm.ku.dk (J.N.); mikkel.sinding@snm.ku.dk (M.-H.S.S.); marcela.velasco@snm.ku.dk (M.S.-V.); mtpgilbert@gmail.com (M.T.P.G.); 3 Department of Natural History, Norwegian University of Science and Technology, University Museum, NO-7491 Trondheim, Norway; mike.martin@ntnu.no; 4 Natural History Museum, University of Oslo, P.O. Box 1172 Blindern, N-0318 Oslo, Norway; 5 Otago Palaeogenetics Laboratory, Department of Zoology, University of Otago, Dunedin 9054, New Zealand; nic.rawlence@otago.ac.nz; 6 65 Springfield Road, Southborough, Tunbridge Wells TN4 0RD, Kent, UK; errolfuller123@btinternet.com; 7 Center for Macroecology, Evolution and Climate, the Natural History Museum of Denmark, University of Copenhagen, Universitetsparken 15, DK-2100 Copenhagen Ø, Denmark; jfjeldsaa@snm.ku.dk; 8 Department of Mathematics and Natural Sciences, Evolutionary Adaptive Genomics, Institute for Biochemistry and Biology, University of Potsdam, Karl-Liebknecht-Str. 24-25, 14476 Potsdam, Germany; michi@palaeo.eu; 9 Faculty of Science and Technology, Bournemouth University, Dorset BH12 5BB, UK; jstewart@bournemouth.ac.uk; 10 Department of Anatomy, University of Otago, 270 Great King Street, Dunedin 9016, New Zealand; michael.knapp@otago.ac.nz

**Keywords:** ancient DNA, extinct birds, mitochondrial genome, museum specimens, palaeogenomics

## Abstract

One hundred and seventy-three years ago, the last two Great Auks, *Pinguinus impennis*, ever reliably seen were killed. Their internal organs can be found in the collections of the Natural History Museum of Denmark, but the location of their skins has remained a mystery. In 1999, Great Auk expert Errol Fuller proposed a list of five potential candidate skins in museums around the world. Here we take a palaeogenomic approach to test which—if any—of Fuller’s candidate skins likely belong to either of the two birds. Using mitochondrial genomes from the five candidate birds (housed in museums in Bremen, Brussels, Kiel, Los Angeles, and Oldenburg) and the organs of the last two known individuals, we partially solve the mystery that has been on Great Auk scholars’ minds for generations and make new suggestions as to the whereabouts of the still-missing skin from these two birds.

## 1. Introduction

Over the past three decades, the field of ancient DNA (aDNA) has grown considerably, from sequencing a small section of mitochondrial DNA from the Quagga, an extinct form of the plains zebra [1], to whole genome sequencing from samples up to 735,000 years old [2]. Ancient DNA has been used to answer and address a diverse range of ecological and evolutionary questions, providing insight into countless species’ pasts, including our own. However, aDNA can also be a useful tool for museums, specifically for species identification and, under suitable circumstances for reconstructing the history of specimens where museum records are insufficient. This study traces the whereabouts of the skins from the last two documented Great Auks using a palaeogenomic approach.

**Figure 1:**
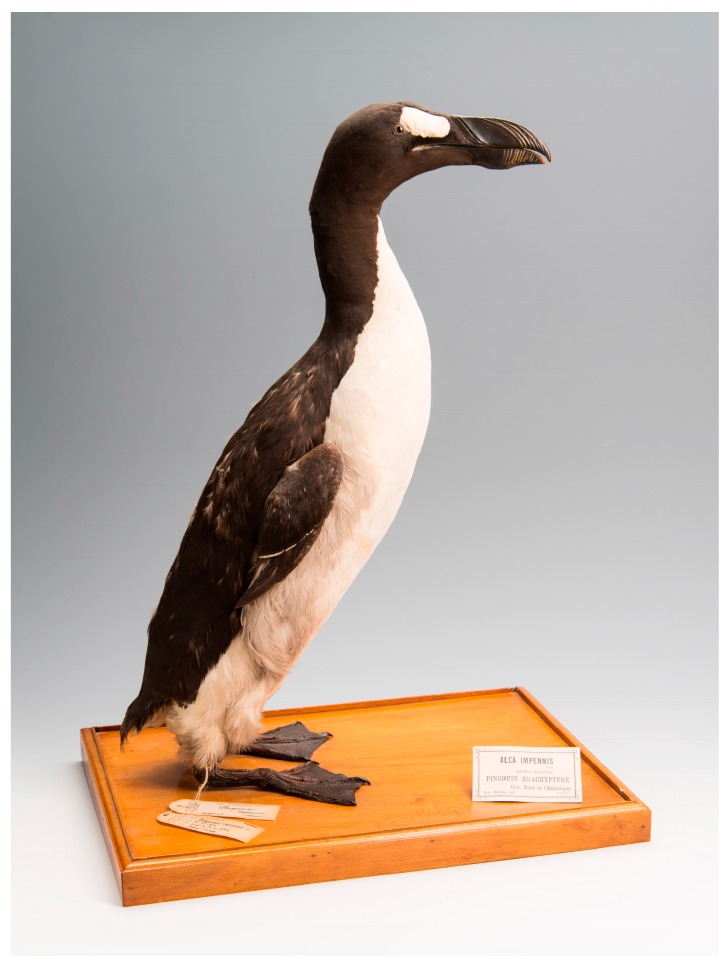
A mounted Great Auk skin, The Brussels Auk (RBINS 5355) (MK135), from the collections at Royal Belgian Institute of Natural Sciences (Credit Thierry Hubin (RBINS)).

The Great Auk (Figure 1), *Pinguinus impennis*, Bonnaterre (1790) (traditionally *Alca impennis*, Linnaeus, 1758), has been described as “*perhaps the most curious of all vanished birds*” [3]. It was a bird whose life and ultimate extinction has generated ongoing interest, with several scholars dedicating their lives to Great Auk research [3,4,5,6,7]. Even now, 173 years after the death of the last two recorded captured individuals, there are still many unanswered questions concerning aspect of its life-history, evolution, and extinction. One such mystery that surrounds the Great Auk is the whereabouts of the skins from the last documented pair. In order to be able to correlate the phenotype of the last birds with genomic information obtained from the well-preserved organs, and in view of the active role that researchers and research institutions played in pushing the Great Auk towards extinction, it is of relevance to be able to trace these skins.

**Figure 2:**
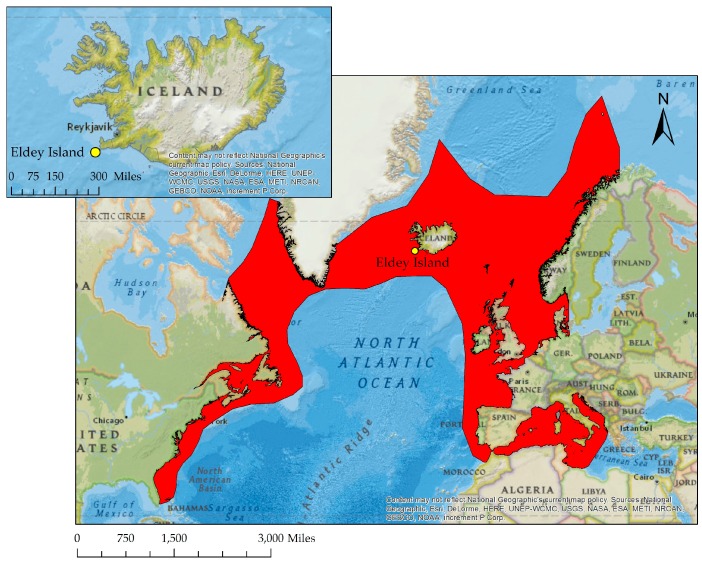
The Great Auk’s breeding range across the North Atlantic, as indicated by the red area and the location of Eldey Island (yellow dot) off the south-west coast of Iceland, the site where the last documented Great Auks were killed. Maps were created using spatial data provided by BirdLife International/IUCN [11] with the National Geographic basemap in ArcGIS (ESRI, Redlands, CA, USA) [12].

Once found in great numbers across the North Atlantic (Figure 2), this flightless bird was heavily hunted for its meat, oil, and feathers. By the start of the 19th century, populations in the North-West Atlantic had been decimated. The last few remaining birds were breeding on the skerries off the south-west coast of Iceland, but with their scarcity increasing, Great Auks were then also sought after as a desirable item for both private and institutional collections [3,5,8,9,10].

From 1830 to 1841, several trips were taken to Eldey Island (Figure 2) where Great Auks were caught, killed, and sold for exhibitions. Following a three-year period of no recorded captures of Great Auks, Carl Siemsen commissioned an expedition to Eldey to search for any remaining birds. Between 2 and 5 June 1844, the expedition reached Eldey Island where two Great Auks were observed amongst smaller birds inhabiting the island. Both Auks were killed and their broken egg discarded. The birds, though, were never to reach Siemsen. The expedition leader sold them to Christian Hansen, who then sold them to the apothecary Möller, in Reykjavik, Iceland. Möller skinned the birds and sent them, as well as their preserved body parts, to Denmark [3,6,7].

The internal organs of these two birds now reside in the Natural History Museum of Denmark. However, the location of the skins of those individuals remains a mystery, despite considerable effort of notable Great Auk scholars to solve it.

Fuller [3] describes in detail the known history of the 80 or so specimens that are still in existence in collections today and concludes: “*Somehow, amid all the frantic Garefowl* [another name for Great Auk] *research of the nineteenth century, they* [the skins] *were lost track of. Several of the surviving stuffed specimens, notably those in Kiel, Bremen and Oldenburg were tentatively identified with them. The most likely candidates, however, are the birds now in Los Angeles and in Brussels*” [3] (p. 85).

Our study compares complete mitochondrial genome (mitogenome) sequences from the five candidate skins (those housed in Bremen, Brussels, Kiel, Los Angeles, and Oldenburg) to the internal organs of the last documented captured Great Auks (stored in Copenhagen) to test which—if any—of Fuller’s candidate skins likely belong to one of the last two individuals.

## 2. Materials and Methods

### 2.1. Sample Information

Specimens from the candidate list proposed by Fuller [3] and the organs from the two 1844 Eldey Island individuals, were sampled using sterile equipment and the appropriate method for sample type, which caused minimal physical damage to the specimen (Table 1). 

### 2.2. DNA Extraction

All lab work prior to polymerase chain reaction (PCR) amplification was carried out in designated aDNA laboratories that adhere to strict aDNA protocols [13]. For each DNA extraction and library build, negative controls were used to check for contamination by exogenous DNA. All post-PCR work on amplified DNA was carried out in separate laboratory facilities.

**Figure 3:**
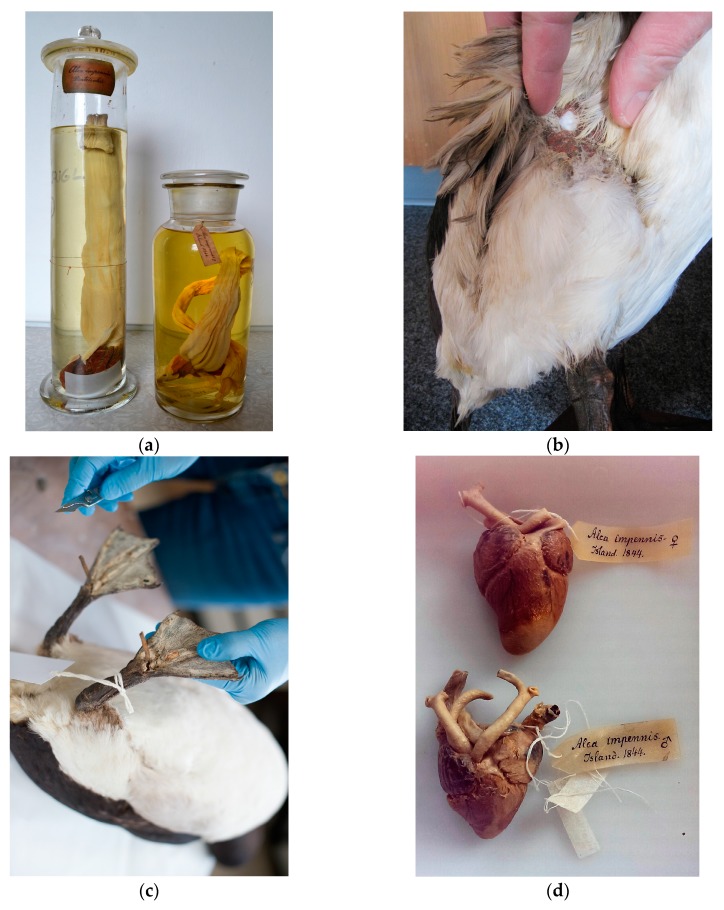
(**a**) Jars containing the oesophagus from the last two individuals killed on Eldey Island. The oesophagus from the larger jar represents that of the individual labelled male (NHMD153069) (MK131). The smaller jar contains the oesophagus from the female bird (NHMD153070) (MK132) (credit. J. Thomas). (**b**) Sampling of The Oldenburg Auk (AVE 8086) (MK133) to remove a section of body tissue for DNA extraction (credit. C. Barilaro, Landesmuseum Natur und Mensch Oldenburg). (**c**) Sampling the toe pad of The Bremen Auk (RKNr. 2392) (MK134) to remove tissue sample (credit M. Stiller, Übersee-Museum Bremen). (**d**) The hearts from the last two documented individuals. The heart from the female individual has been sampled for this study (top) (NHMD153070) (LastGA2_Heart) (credit Natural History Museum of Denmark).

Genomic DNA was extracted from the oesophagus (Figure 3a), skin (Figure 3b), toepad tissue (Figure 3c), and feathers using a modified version of Dabney et al. [14] in which the initial digestion was carried out following the protocol by Gilbert et al. [15]. This digestion buffer is better suited to extraction from these tissues types than the Dabney et al. [14] digestion buffer, which was optimised for DNA extraction from bone. Subsequent DNA purification and elution was conducted following the approach described by Dabney et al. [14]. Genomic DNA was extracted from the heart tissue (Figure 3d) using the protocol by Campos et al. [16].

### 2.3. Data Generation

Single stranded libraries were constructed for all samples, except LastGA2_Heart, following Gansauge & Meyer [17], with modifications as described by Bennett et al. [18], as this allowed for targeting of the smallest fragments of DNA, typical of highly degraded specimens. For LastGA2_Heart, the protocol described by Meyer & Kircher [19] was used. Enrichment for complete mitogenomes was performed using MYcroarray MYbaits, following the manufacturer’s manual v2.3.1 [20] on all samples except MK138 and LastGA2_Heart. Samples were sequenced on Illumina platforms (HiSeq and MiSeq) by New Zealand Genomics Limited, Otago Branch, or the Danish National High-Throughput DNA Sequencing Centre.

### 2.4. Read Processing

Processing of raw sequence data was facilitated by the PALEOMIX v1.2.5 pipeline [21], which performs adapter trimming, read mapping to a reference genome, and quality-based filtering. Low-quality bases and adapter sequences were trimmed from the 3’ ends of DNA reads with the software AdapterRemoval v2.1.7 [22,23] using a mismatch rate of 0.333 (command-line option—mm 3). Paired end reads overlapping by at least 11 base pairs (bp) were collapsed into a single read with re-calibrated base quality scores. Trimmed reads shorter than 25 bp were discarded.

Mapping to the Great Auk reference mitogenome (GenBank: KU158188.1) [24] was performed with Burrows–Wheeler Aligner (BWA) v0.5.10 [25] with seeding deactivated and otherwise default settings. PCR duplicates were removed with the MarkDuplicates function within Picard v1.82 [26] and the rmdup function within the software SAMtools [27]. Collapsed reads were filtered using a script included with PALEOMIX. Reads with mapping quality (MAPQ) scores <20 were removed from further analysis. Local realignment of reads misaligned to the reference mitogenome was performed with the RealignerTargetCreator and IndelRealigner tools included in the software Genome Analysis Toolkit (GATK) v3.6.0 [28]. The pipeline also utilised MapDamage2 [29] to recalibrate base qualities of aligned sequence reads in each sequencing library in order to remove the residual aDNA damage patterns. The UnifiedGenotyper algorithm within GATK v3.6.0 was used to determine haploid genotypes within individual samples.

A relaxed and strict filtering system was used to create consensus sequences and alignments from the processed data. In the first stage of filtering, both systems used VCFtools [30] to filter genotypes from the final alignment when their genotype quality scores were less than 30. For the relaxed alignment, the per-individual read depth was set to only include bases with a minimum of 3-fold coverage. Bases called for the consensus sequence had to be present at a frequency higher than 33%. To be included in the final alignment, no more than 33% of bases could be missing from the consensus sequence of an individual.

For the strict settings, the per-individual read depth was set to only include bases with at least 10-fold coverage. Geneious v-10.1.3 [31] was used to filter bases so that the majority base was present in more than 90% of reads. For an individual to be included in the final alignment, no more than 20% of sites could be missing from the individual’s consensus sequence. 

A custom script was used to convert the filtered Variant Call Format (VCF) file into a multiple sequence alignment in FASTA format.

Following read processing, the data was aligned using Seaview v4.0 [32] with the algorithm *Muscle -maxiters2 -diags.* The alignment was manually checked for errors using BioEdit v7.2.5 [33], and Tablet v-1.16.09.06 [34] was used to view the rescaled Binary Alignment Map (BAM) file for each sample.

MEGA v-7.0.21 [35] was used to generate a pairwise distance table for all sequenced individuals. Phylogenetic relationships between the individuals were reconstructed and visualized using a maximum-likelihood approach as implemented in MEGA v-7.0.21 [35]. jModelTest v-2.1.10 [36,37] was used to determine the most suitable nucleotide substitution model, which was a Hasegawa–Kishino–Yano (HKY) [38] model. Initial trees for the heuristic search were obtained by applying Neighbour-Joining methods to a matrix of pairwise distances estimated using the maximum composite likelihood approach. Branch lengths are measured in number of substitutions per site. All positions containing gaps and missing data were removed. Phylogenies were reconstructed from 500 bootstrap pseudoreplicates to evaluate branch support.

## 3. Results

Mitogenome sequence data was obtained from all candidate specimens as well as from the two oesophagi of the last Great Auks. Unique coverage of the mitogenomes for these samples ranged from 6.2× to 288.6× (Table 2). As DNA extracted from the oesophagus of the female last Great Auk (MK132) yielded only a low coverage, poor quality mitogenome assembly, DNA from the heart of the same individual was also sequenced. This yielded a high coverage (430×) mitogenome, which was used in all further analyses.

With the sequence data from the heart of the female last Great Auk (LastGA2_Heart), the alignment of all sequences assembled under the relaxed rules had a length of 15,790 bp after sites not covered by all consensus sequences were removed. For the strict alignment, MK138 did not meet criteria set by the strict filtering settings as more than 20% sites were missing. With this individual removed, we obtained a strict alignment length of 13,475 bp.

**Figure 4:**
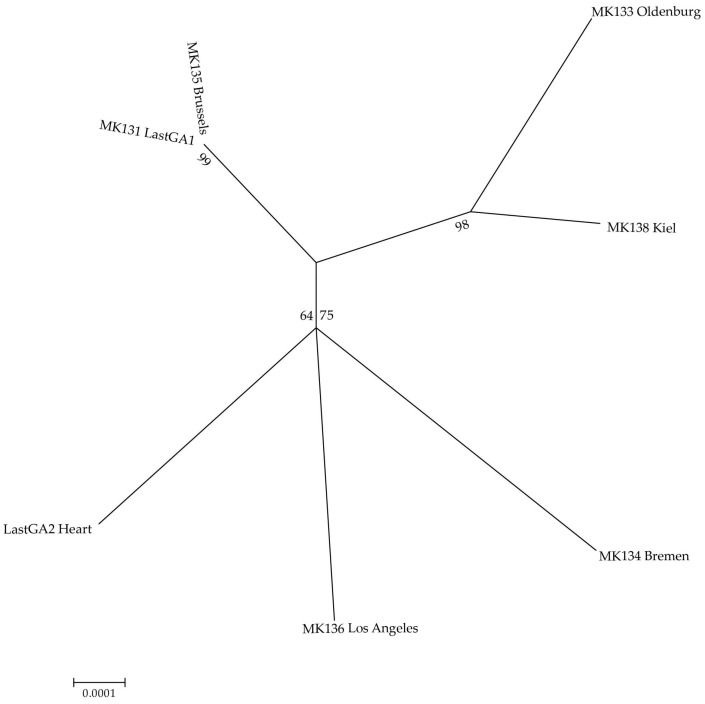
Maximum liklihood reconstruction of phylogenetic relationships between individuals, under the relaxed filtering settings. Branch labels are bootstrap support values for the respective sample. Evolutionary analyses were conducted in MEGA7 [35].

The pairwise distance matrix (Table 3) shows that the consensus sequence obtained from sample MK131, the oesophagus of the male, is identical to the consensus sequence obtained from MK135, The Brussels Auk. No other consensus sequences match. LastGA2_Heart, the female last Great Auk, groups with MK136 and MK134 in the maximum likelihood phylogeny (Figure 4), but there are 18 and 20 well-supported differences between the consensus sequences, respectively. Analysis presented here was generated using data from the relaxed filtering settings, but results were consistent with data from the strict filtering system. Thus, only the male last Great Auk has a corresponding DNA match among the candidate skin samples identified by Fuller [3].

## 4. Discussion

The genetic analyses presented here help to partially resolve the mystery of the missing skins of the last two Great Auks. They provide evidence of matching mitochondrial genomes for the internal organs of the last male Great Auk held at the Natural History Museum of Denmark in Copenhagen and the Great Auk skin held at the Royal Belgian Institute of Natural Sciences, Brussels (Figure 1). Mitochondrial DNA cannot always be unambiguously used in identification of individuals. However, in a broader study of forty one Great Auk mitogenomes from across their range, Thomas et al. (in prep) [39], found that mitochondrial diversity in Great Auks remained high right up to their demise, with no other individuals found to have the same mitochondrial haplotype. Together with the information from the historical record, the match between the internal organs and The Brussels Auk therefore appears to be more than just a coincidence.

There are around 80 known mounted Great Auk skins in museums worldwide. However, the majority can be ruled out of any speculation that they belonged to the last pair due to their history (for example, if they were collected before 1844). Those tested in the current study were placed on the candidate specimen list due to several factors that led Fuller, as well as other experts like the University of Copenhagen Professor Japetus Steenstrup (dubbed ‘Father of Garefowl History’ by Grieve, 1885), and Grieve [4], to suspect that they originated from the 1844 Eldey pair. Details such as when and where they were acquired, from whom (i.e., the dealer), and suggestions by renowned Great Auk scholars made the birds in Bremen, Brussels, Kiel, Los Angeles, and Oldenburg the top candidates [3].

In the museum industry, accurate records and archiving are obviously of high priority, with labels and registers providing vital information about the specimens [40,41,42]; it therefore seems unexpected that the two bird skins could have been “lost”. However, at the time, their significance as the final remnants of the species was not recognised. The story of the ending of these individuals lives is well documented due to the efforts of English naturalist John Wolley and Cambridge University Professor Alfred Newton, who travelled to Iceland in the late 1850s and spoke directly with those who were part of the 1844 Eldey Island voyage (details from Wolley’s notebook ‘Garefowl books’ published in Newton, 1861 [7]). What happened once the skins and their organs reached Denmark, on the other hand, is poorly recorded and remains speculative [3].

In the archives of Cambridge University are the fragments of information that Newton learned of the birds. On notes dated 1861, it was recorded that Professor Reinhardt of the Royal Museum (Copenhagen) believed the skins and their organs had been purchased for the museum by Professor Eschricht of the University of Copenhagen. He is said to have taken the skins to the Congress of German Naturalists in Bremen in the autumn of 1844 [3].

The connection with the skins and the Congress in Bremen could be what led Steenstrup to inform Grieve of his suspicions that the specimen at the museum in Bremen (MK134) was indeed one of the last birds [4]. Yet, this bird was bought by the museum at the time of the Congress from the Hamburg dealer Salmin, not Eschricht. Therefore, while the possibility may be there for Salmin to have first had the bird from Eschricht and then sold it on, it is also likely that it was a bird he had in his stocks prior to 1844 [3]. This study shows The Bremen Auk is not a match with either of the organs from the last pair, suggesting that it did indeed come from an earlier raid of Eldey.

The specimen in Kiel, the Schleswig–Holstein Auk (MK138), was purchased in 1844. With such a suggestive purchase date it is a contender in the mystery [3]. Professor Steenstrup was quoted by Grieve as saying, “*If really purchased in 1844, it might perhaps be the second of these two Garefowls got in 1844, but traditionally I never heard that mentioned*” [4] (Grieve Appendix p. 13 [4]). Our study shows this specimen was not a match, so Steenstrup was correct in his belief.

With regard to The Oldenburg Auk (MK133), this specimen was once regarded by nineteenth century scholars as belonging to one of the last birds. However, the records for this bird shows it was obtained prior to 1844 and is therefore ruled out [3]. It was tested in this study due to the suggestions of these early researchers but was not a match.

The history of The Brussels Auk (MK135) and Dawson Rowley’s Los Angeles Auk (MK136) can be traced back to 1845 when they were said to be in the hands of a well-known, and well connected, Great Auk dealer, Israel of Copenhagen. Israel is known to have had excellent links with Iceland and spent his winters in Copenhagen and his summers in Amsterdam [3]. Fuller suggests that perhaps Israel, if he did not receive them direct from Iceland, purchased the birds in Bremen from Eschricht. The birds have a detailed history, passing through the hands of several dealers. From Israel, they were bought by Lintz, a Hamburg merchant, and in 1845 were sold on to the Amsterdam branch of the dealer, Frank. In Newton’s notes at Cambridge it was recorded that Frank believed the two skins he bought were from the last pair. The Brussels Auk was purchased in 1847 by Viscount Bernard Du Bus Ghisignies, director of the Brussels Museum [3]. The history of The Brussels Auk therefore strongly supports our positive match with MK131.

If the bird in Brussels, which came from Israel of Copenhagen, is from one of the last birds, then this would suggest that the second bird he had would also be from Eldey in 1844 and therefore be a positive match with the second set of organs. Israel’s second bird has an even longer story than that of MK135, but it now resides in the Natural History Museum of Los Angeles County [3]. This specimen, Dawson Rowley’s Los Angeles Auk (MK136), was tested, and the results showed it did not match LastGA2_Heart. With this negative result, we can only speculate which of the remaining untested birds could be identified as the second individual.

A possible scenario to explain the mismatch between Dawson Rowley’s Los Angeles Auk (MK136) and the internal organs from the Natural History Museum of Denmark involves a mix up of skins. Dawson Rowley’s Los Angeles Auk, was once one of two Great Auks owned by George Dawson Rowley. During the 1930s, they were passed to Captain Vivian Hewitt who owned two additional specimens. The four specimens are currently held in Cardiff, Birmingham, Los Angeles, and Cincinnati. At Hewitt’s death, his collection had been put under the control of Spink and Son Ltd., a London dealer, who offered them for sale. While organising Hewitt’s affairs, the four birds were mixed up. The identity of the birds now in Birmingham and Cardiff could be easily resolved, but those now in Los Angeles and Cincinnati are harder to determine. It is thought that their identities could be determined from annotated photographs taken in 1871 by George Dawson Rowley when they were in his possession [3]. However, we speculate that their identities were not correctly resolved and that perhaps the bird in Cincinnati was the original bird from Israel of Copenhagen. If this were the case, then it would explain why the Los Angeles bird fails to match with either of the last Great Auk organs held in Copenhagen.

In summary, we suggest that The Brussels Auk is the skin from the last male Great Auk killed on Eldey Island in June 1844. The skin of the female killed at the same time remains unaccounted for, but a common history with The Brussels Auk makes the skin currently held at Cincinnati Museum of Natural History and Science, a likely candidate. A re-evaluation of the historical records may reveal further candidate skins amongst those currently held in museums around the world.

## 5. Conclusions

Ancient DNA has been used to evaluate museum collections in the past, albeit usually for taxonomic identification of unidentified or misidentified accessions. Our study shows an alternative use of the technology. It demonstrates the utility of molecular tools and advanced sequencing to contribute to questions, which are not primarily biological or molecular but rather historical in nature. The unraveling of the mystery surrounding the whereabouts of the skins of the last two Great Auks represents a fascinating element in the story of extinction and human involvement in that process.

## Figures and Tables

**Table 1:** Sample information. Lab ID number used during laboratory and analysis process. Mount name and description given by Fuller and its number in various published lists of Great Auk mounts [3]. Origin and date information as noted by Fuller [3]. Institution information relating to the present location of specimen and the curator/sample collector name.

Lab ID	Bird Name, Number & Description	Origin & Date	Institution	Curator/Collector	Institution Number	Sample Type/Sampling Method
MK131	Last Great Auk 1 Oesophagus (male)	Eldey Island, Iceland. Date: June 1844	Natural History Museum of Denmark. Copenhagen, Denmark	J. Fjeldså/ J. Thomas	NHMD 153069	Oesophagus. Tissue cut from end of oesophagus.
MK132	Last Great Auk 2 Oesophagus (female)	Eldey Island, Iceland. Date: June 1844	Natural History Museum of Denmark. Copenhagen, Denmark	J. Fjeldså/ J. Thomas	NHMD 153070	Oesophagus. Tissue cut from end of oesophagus.
MK133	The Oldenburg Auk Fuller: Bird no. 47, Grieve: no. 57, Hahn: no. 77 Adult in summer plumage	Iceland. Probably Eldey. Date: Unknown	Landesmuseum Natur und Mensch Oldenburg. Germany	C. Barilaro	AVE 8086	Body tissue. Tissue cut from body of bird under wing.
MK134	The Bremen Auk Fuller: Bird no. 36, Grieve: no. 10, Hahn: no. 71 Adult in summer plumage	Unknown. Probably Eldey. Date: Unknown	Übersee-Museum Bremen. Germany	M. Stiller	RKNr. 2392	Toepad tissue. Tissue cut from feet.
MK135	The Brussels Auk Fuller: Bird no. 3, Grieve: no. 15, Hahn: no. 6 Adult in summer plumage	Probably Eldey Date: Unknown perhaps June, 1844	Institut Royal des Sciences Naturelles de Belgique. Brussels, Belgium	G. Lenglet	RBINS 5355	Toepad tissue. Tissue cut from feet
MK136	Dawson Rowley’s Los Angeles Auk Fuller: Bird no. 73, Grieve no. 13, Hahn: no. 5 Adult in summer plumage, said to be female	Iceland. Probably Eldey. Date: Unknown perhaps June, 1844	Natural History Museum of Los Angeles County. USA	K. Garett	LACM 76476	Feather. Feathers plucked from body of bird.
MK138	The Schleswig-Holstein Auk Fuller: Bird no. 42, Grieve: no. 31, Hahn: no. 74 Adult in summer plumage	Unknown Date: Unknown	Zoologisches Museum der Christian-Albrechts Universität zu Kiel. Germany	D. Brandis/ L. Rosotta	cat. No. A0585	Toepad tissue. Tissue cut from feet.
LastGA2_Heart	Last Great Auk 2 Heart (female)	Eldey Island, Iceland. Date: June 1844	Natural History Museum of Denmark. Copenhagen, Denmark	J. Fjeldså/ J. Haile	NHMD 153070	Heart. Tissue cut from aorta.

**Table 2:** Read processing results for all samples.

Sample	GenBank Accession Number	Number of Reads	Number of Unique Reads Mapping to Reference Mitogenome	Estimated Coverage from Unique Hits	Relaxed Settings Sequence Length (bp ^1^)	Strict Settings Sequence Length (bp)
MK131	MF188883	300754 (read pairs)	30,297	74.40	16,001	15,067
MK132	NA	550631 (read pairs)	2366	6.23	13,267	3312
MK133	MF188884	429392 (read pairs)	8750	23.04	16,251	14,240
MK134	MF188885	343766 (read pairs)	86,325	288.62	16,607	16,526
MK135	MF188886	579992 (read pairs)	27,767	88.90	16,554	16,356
MK136	MF188887	563635 (read pairs)	24,401	67.83	16,330	15,833
MK138	MF188888	10796460 (SE ^2^ reads)	2799	9.76	16509	7866
LastGA2_Heart	MF188889	957970612 (SE reads)	121,886	430.09	16,698	16,649

^1^ Base pairs (bp); ^2^ Single End (SE).

**Table 3:** Pairwise distance matrix. Estimates of evolutionary divergence between sequences generated using the relaxed settings. The number of base differences per sequence from between sequences are shown. All positions containing gaps and missing data were removed, leaving a total of 15,790 positions in the final dataset. Evolutionary analyses were conducted in MEGA7 [35].

	MK131	MK133	MK134	MK135	MK136	MK138	LastGA2_Heart
MK131_LastGA1							
MK133_Oldenburg	17						
MK134_Bremen	18	23					
MK135_Brussels	0	17	18				
MK136_LA	16	23	20	16			
MK138_Kiel	14	11	20	14	20		
LastGA2_Heart	16	23	20	16	18	20	
